# Behaviors of Electromagnetic Wave Propagation in Double-Walled Carbon Nanotubes

**DOI:** 10.3390/ma14154069

**Published:** 2021-07-21

**Authors:** Ayse Nihan Basmaci

**Affiliations:** Vocational School of Technical Sciences, Tekirdag Namik Kemal University, 59030 Tekirdag, Turkey; anbasmaci@nku.edu.tr

**Keywords:** electromagnetic wave propagation, carbon nanotubes, double-walled carbon nanotubes, Maxwell equations

## Abstract

In this study, behaviors of electromagnetic wave propagation in a double-walled carbon nanotube (DWCNT) are investigated theoretically. For this purpose, the effects of carbon nanotube’s inner and outer tubes’ material property parameters (*μ*, *ε*) on electromagnetic wave propagation are discussed. The effects of interaction between the carbon nanotube’s inner and outer tubes on the electromagnetic wave propagation are defined. Nonlocal effects of the DWCNT on electromagnetic wave propagation are examined. Besides, the electromagnetic wave propagation frequencies are specifically investigated, taking the DWCNT’s nonlocal effects and material property parameters (ε, *µ*) into account. When the wavenumber, k, is greater than 1.8 × 10^10^, the frequencies of the fundamental mode and the second mode converge to 3.554 × 10^8^ Hz. Additionally, the electromagnetic wave propagation frequencies decrease with the increase of the DWCNT’s nonlocal parameter (*ν*) and decrease with material parameter (*D*).

## 1. Introduction

Owing to the developments in nanoscale engineering, the requirement of carbon nanotubes (CNTs) increases more and more [1]. Graphene and CNTs have been mostly used in the areas of stealth technology and light-weighted vehicle design. Some works in the literature are related to the advantages of CNTs in terms of both mechanical and electrical aspects [2,3,4,5,6,7]. Nanoscale structures have very good electrical, magnetic, mechanical, and thermal properties [8,9,10,11]. By taking advantage of these properties, micro-electromechanical structures (MEMS) and nano-electromechanical structures (NEMS) are produced [12,13]. Multi-walled carbon nanotubes (MWCNTs) ranging in diameter from 8 to 15 nm are obtained from aluminum particles coated with polyurethane [14]. Additionally, scanning electron microscopy images of these MWCNTs, including different Al contents, are investigated.

Composites and nanocomposites are basic examples of CNT-based structures [14,15,16,17,18,19,20]. In order to achieve electromagnetic wave absorption, special coatings called radar absorption materials (RAM) including multi-walled carbon nanotubes (MWCNT) are mostly used in the defense industry [21,22].

The behaviors of electromagnetic wave propagation in waveguides have been investigated [23,24]. The design methodologies of both phononic and photonic structures are defined with the electromagnetic and acoustic wave equations. On this basis, the behaviors of wave propagation in phononic structures have been investigated [25,26,27]. In a planar lightwave circuit (PLC), optical wave guiding can be achieved by dividing the waveguide in multiple ways. Likewise, several techniques have been developed to achieve optical waveguiding related to the photonic-crystalline structures [28,29,30,31,32].

The electromagnetic wave propagation behaviors of a photonic waveguide divided into four sections arranged side by side, and the energy of the moving wave in each part of the waveguide are examined [33]. In [33], electromagnetic wave propagation behavior of photonic crystals at macroscale is investigated, unlike in [33] where electromagnetic wave propagation is investigated in double-walled carbon nanotubes with inner and outer tubes interacting with each other at nanoscale. The frequencies of electromagnetic wave propagation occurring in these different structures are examined similarly in both studies.

The Maxwell equations are solved using different numerical methods to investigate the behaviors of electromagnetic wave propagation in several structures [34,35,36,37].

Three different methods can examine nanoscale structures. The first of these methods is the experimental analysis method. With the possibilities offered by today’s technology, only a limited number of processes can be performed with the experimental analysis method used in the analysis of CNTs. Examples of these processes are the CNT stress test and electromagnetic shielding analysis [38,39]. It is not possible to perform more complex analysis, such as wave propagation analysis using the experimental analysis method. The second method used in the investigation of nanoscale structures is atomic simulations (molecular dynamics) [40,41]. The atomic simulations method can give optimum results in the analysis of small-scale systems for small time intervals. It is necessary to have a good software infrastructure in order to use this method. In atomic simulation processes, each bond between each atom of the CNTs is taken into account. Therefore, it is very difficult to reach the exact solution in atomic simulation processes. Using good software in atomic simulation processes is a requirement to get high accuracy results. The third method used in the investigation of nanoscale structures is model-based methods. Model-based methods include finite element and finite difference methods. These methods are generally used in solving complex problems [42,43,44,45].

In this study, the nonlocal theory of Eringen, one of the most well-known models, is used. This method is preferred because it allows easy access to the exact solution. The success of the nonlocal theory is because of the comparison of this method with the method of atomic simulations. In order to understand the accuracy of the results obtained using the non-local theory, the results obtained by the nonlocal theory were compared with the results obtained by the method of atomic simulations. Both results are obtained to be very close to each other [46]. The basic principle of the nonlocal theory is simply that two atoms that are not adjacent to each other affect each other [47,48]. Considering that CNTs are very small in size, it is clear enough that the most ideal method to be used for the analysis of these structures is the nonlocal theory.

Nonlocal effects of a carbon nanotube on electromagnetic wave propagation are discussed, and the difference between effects of local and nonlocal states of nanostructures on the wave propagation is investigated [49,50,51,52,53]. In addition, there are some studies on electromagnetic wave dispersion behaviors and electromagnetic wave velocities, such as phase velocities and group velocities of carbon nanotubes [54,55,56]. In this study, the electromagnetic wave propagation frequencies obtained when the electromagnetic waves propagating in the inner and outer tubes of the DWCNT are in phase are called Mode 1 frequencies. The electromagnetic wave propagation frequencies obtained when the electromagnetic waves propagating in the inner and outer tubes of the DWCNT are anti-phase are called Mode 2 frequencies. In this study, unlike other studies in the literature, Mode 2 frequencies related to electromagnetic wave propagation in DWCNT tubes have been examined. Although there are many carbon nanotube studies in the literature, the number of works that focus on the effects of electromagnetic interaction between inner and outer tubes of a double-walled carbon nanotube (DWCNT) on electromagnetic wave propagation is quite low. In this study, electromagnetic wave propagation frequencies in a DWCNT, whose inner and outer tubes have different material property parameters (*µ, ε*), are examined. In order to obtain electromagnetic wave propagation frequencies, nonlocal effects of the DWCNT and the effects of electromagnetic (EM) interaction between DWCNT’s tubes are also taken into account. Unlike other studies in the literature, this study also investigates electromagnetic wave dispersion relation between different wavenumbers and electromagnetic wave propagation frequencies obtained for different material properties of the DWCNT’s inner and outer tubes.

## 2. Theoretical Analysis

**Figure 1:**
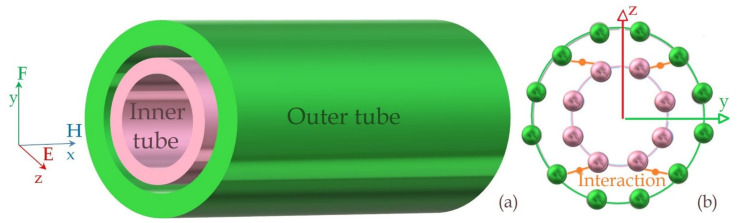
(**a**) EM wave propagation in the DWCNT, (**b**) EM interaction between DWCNT’s inner and outer tubes.

Behaviors of electromagnetic wave propagation in a double-walled carbon nanotube (DWCNT) are investigated. Figure 1 illustrates the DWCNT. There is electromagnetic interaction defined with an interaction coefficient of *α* between DWCNT’s inner and outer tubes. As also seen from the figure, the inner and the outer tubes of the DWCNT have different material property parameters (*μ*, *ε*), and the electromagnetic wave propagates lossless along its *x*-axis.

In a source free, linear, isotropic, and homogenous region, Maxwell equations are [36]:

(1) $$\nabla \times \vec{\;E}=-j\omega \mu \vec{H}$$

(2) $$\nabla \times \vec{\;H}=j\omega \varepsilon \vec{E}$$

where $j=\sqrt{-1}$, *µ* is the permeability, *ε* is the permittivity, $\vec{E}$ is the electrical field, and $\vec{\;H}$ is the magnetic field. Using Equations (1) and (2), Equation (3) is obtained:

(3) $$\nabla \times \left(\nabla \times \vec{H}\right)=\nabla \left(\nabla \cdot \vec{H}\right)-\nabla^{2}\vec{H}=\nabla \times \left(-\mu \frac{\partial \vec{H}}{\partial t}\right)$$

By solving Equation (3), a partial differential equation related to the electromagnetic wave propagation depending on time and position is obtained:

(4) $$\frac{1}{\mu \varepsilon }\frac{\partial^{2}H_{x}}{\partial x^{2}}-\frac{\partial^{2}H_{x}}{\partial t^{2}}=0$$

where *H_x_* represents the magnetic field in the x-direction.

According to the nonlocal theory, the material property parameters (*μ*, *ε*) of any point on the carbon nanotube, determined as the reference point, affect the material property parameters (*μ*, *ε*) of all local points adjacent to the reference point. The material property parameters (*μ*, *ε*) of the reference point also affect the material property parameters (*μ*, *ε*) of all nonlocal points that are not adjacent to the reference point. The electromagnetic wave equation of a single-walled carbon nanotube (SWCNT) can be written in the following form [47,48]:

(5) $$D\frac{\partial^{2}H_{x}}{\partial x^{2}}=\left[1-{\left(e_{0}a\right)}^{2}\frac{\partial^{2}}{\partial x^{2}}\right]\frac{\partial^{2}H_{x}}{\partial t^{2}}$$

where *D =* 1/(*με*) represents a material parameter, “a” is an internal characteristic length, “$e_{0}$” is a constant, and $e_{0}a=\nu$ represents the nonlocal coefficient.

Electromagnetic wave equations of the DWCNT whose inner and outer tubes are electromagnetically coupled to each other with an interaction coefficient effect of *α* can be written:

(6) $$D_{1}\frac{\partial^{2}H_{1}}{\partial x^{2}}=\left(1-{\left(e_{0}a\right)}^{2}\frac{\partial^{2}}{\partial x^{2}}\right)\frac{\partial^{2}H_{1}}{\partial t^{2}}-\alpha \left(H_{1}-H_{2}\right)$$

(7) $$D_{2}\frac{\partial^{2}H_{2}}{\partial x^{2}}=\left(1-{\left(e_{0}a\right)}^{2}\frac{\partial^{2}}{\partial x^{2}}\right)\frac{\partial^{2}H_{2}}{\partial t^{2}}-\alpha \left(H_{2}-H_{1}\right)$$

where indices “1” refers to the DWCNT’s inner tube and indices “2” refer to the DWCNT’s outer tube. *D_1_*, *D_2_*, *H_1_*, and *H_2_* represent the inner tube’s material parameter, the outer tube’s material parameter, the inner tube’s magnetic field, and the outer tube’s magnetic field, respectively. Equations (6) and (7) are electromagnetic wave equations of the DWCNT’s inner and outer tubes. Displacement of electromagnetic waves is defined as $H\left(x,t\right)=he^{j\left(\omega t-kx\right)}$ where *h,* k, and *ω* represent travelling wave, wave number, and electromagnetic wave propagation frequency, respectively.

Integrating $H\left(x,t\right)=he^{j\left(\omega t-kx\right)}$ into Equations (6)–(10) are obtained:

(8) $$P_{1}=-D_{1}k^{2}+\omega^{2}+\alpha +\omega^{2}\nu^{2}k^{2}$$

(9) $$P_{2}=-D_{2}k^{2}+\omega^{2}+\alpha +\omega^{2}\nu^{2}k^{2}$$

(10) $$P_{12}=P_{21}=-\alpha$$

By setting the determinant of the coefficients matrix obtained from Equations (8)–(10) equal to zero, Equation (11) is obtained as in the following form:

(11) $$\left|\left[\begin{array}{cc} P_{1} & P_{12}\\ P_{21} & P_{2} \end{array}\right]\right|=0$$

By solving Equation (11), frequencies can be obtained as in the following forms:

(12) $$\omega_{1}\left(k\right)=\sqrt{\frac{\frac{D_{1}k^{2}}{2}+\frac{D_{1}k^{4}v^{2}}{2}+\frac{D_{2}k^{4}v^{2}}{2}+\frac{D_{2}k^{2}}{2}-\alpha k^{2}v^{2}-\alpha -\frac{\sqrt{D_{1}{}^{2}k^{4}-2D_{1}D_{2}k^{4}+D_{2}{}^{2}k^{4}+4\alpha^{2}}}{2}}{k^{4}v^{4}+2k^{2}v^{2}+1}}$$

(13) $$\omega_{2}\left(k\right)=\sqrt{\frac{\frac{D_{1}k^{2}}{2}+\frac{D_{1}k^{4}v^{2}}{2}+\frac{D_{2}k^{4}v^{2}}{2}+\frac{D_{2}k^{2}}{2}-\alpha k^{2}v^{2}-\alpha +\frac{\sqrt{D_{1}{}^{2}k^{4}-2D_{1}D_{2}k^{4}+D_{2}{}^{2}k^{4}+4\alpha^{2}}}{2}}{k^{4}v^{4}+2k^{2}v^{2}+1}}$$

where the cut-off frequency is defined as $\omega_{cut-off}=\omega_{2}\left(0\right)$.

Phase velocities, v_phase_ and group velocities, v_group_ regarding the electromagnetic wave propagation in the DWCNT are obtained as follows:

(14) $$v_{\mathrm{phase}}=\frac{\omega }{k}$$

(15) $$v_{\mathrm{group}}=\frac{\partial \omega }{\partial k}$$

## 3. Results

**Figure 2:**
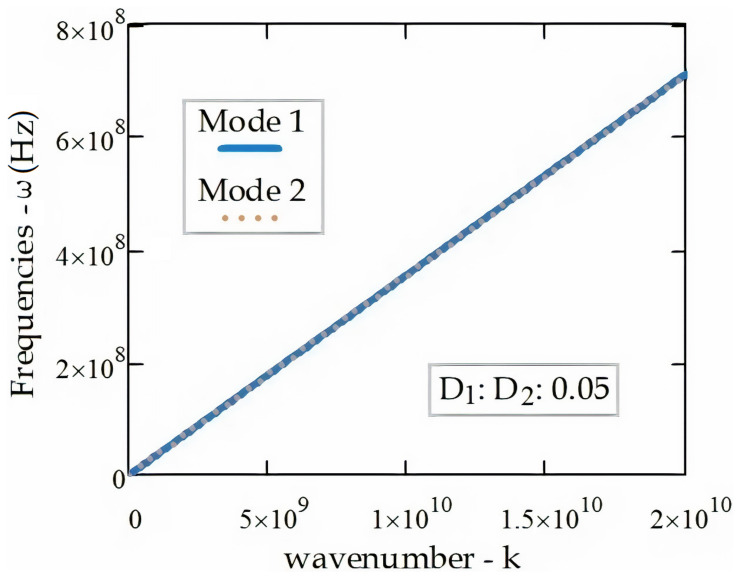
EM wave dispersion relation (k, ω) in the SWT.

Figure 2 illustrates the dispersion relation between wavenumbers and frequencies of electromagnetic wave propagation in a single-walled tube (SWT). It should be noted that the Mode 1 and Mode 2 frequencies are coincident.

The EM wave dispersion relation between wavenumbers and frequencies of the electromagnetic wave propagation in the DWCNT is examined by using Equations (12) and (13). The effects of the DWCNT’s material property parameters (*μ*, *ε*) on the electromagnetic wave propagation are also investigated.

**Figure 3:**
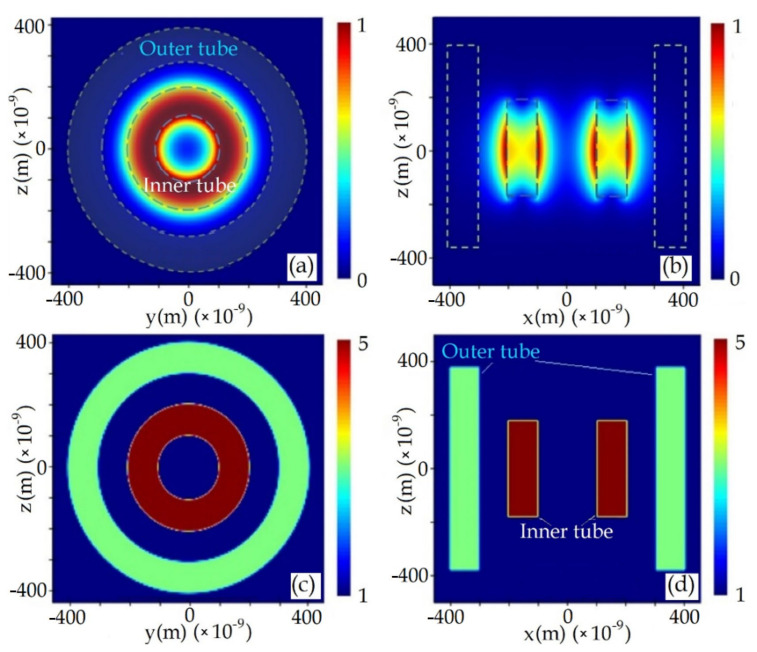
DWCNT’s EM field distribution maps’, (**a**) y-z plane view, (**b**) x-z plane view, and DWCNT’s material property parameters’ (*µ, ε*) distribution maps’, (**c**) y-z plane view, (**d**) x-z plane view.

The DWCNT whose inner and outer tubes are in electromagnetic interaction, and whose inner and outer tube’s material property parameters (*μ*, *ε*) are different is investigated in terms of electromagnetic field distribution and material property parameters (*μ*, *ε*) distribution. Figure 3 shows electromagnetic field distribution and material property parameters (*μ*, *ε*) distribution maps which are obtained by the program ANSYS Lumerical, ANSYS Canada Ltd., Vancouver, BC, Canada. In this section, all plots except Figure 3 are obtained by means of program MathCAD 14, Parametric Technology Corporation, Needham, MA, USA.

**Figure 4:**
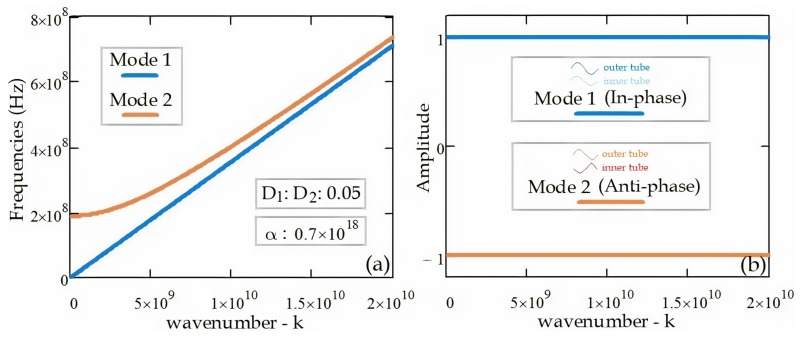
(**a**) EM wave dispersion relation (k, ω), (**b**) EM wave amplitudes in the DWT.

Figure 4a depicts the EM wave dispersion relation (k, ω) between wavenumbers and frequencies of electromagnetic wave propagation in the double-walled tube (DWT). Mode 1 and Mode 2 frequencies of the electromagnetic wave propagation in the DWT converge as the wavenumber, k increases. Mode 2 frequencies start from the cut-off frequency, and electromagnetic wave propagation does not occur below the cut-off frequency value. As can be seen from Figure 4b, the electromagnetic waves in the tubes of the DWT are in-phase at Mode 1 frequencies and in anti-phase at Mode 2 frequencies. There is a 180 phase shift between the electromagnetic waves at Mode 2 frequencies where the electromagnetic waves are in the anti-phase.

**Figure 5:**
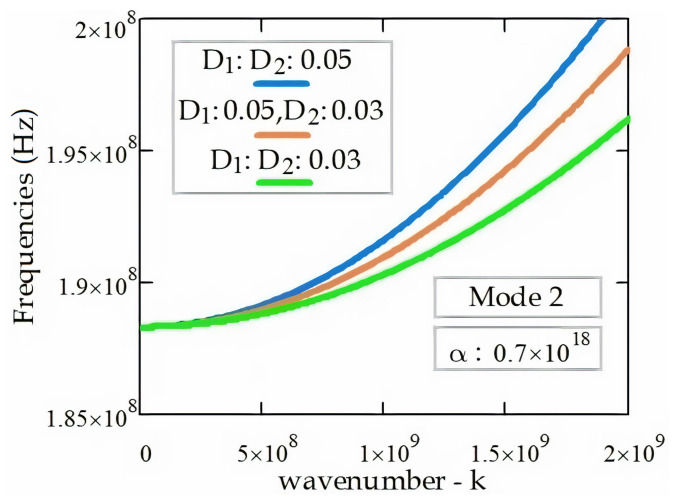
The EM wave dispersion relation (k, ω) in the DWT according to the different material parameters.

Using *D* = 1/(*με*) and the permeability and permittivity values from [17], the material parameter, *D*, is obtained as 0.05 approximately. The behaviors of electromagnetic wave propagation in the DWT having a material parameter of *D_1_* for the inner tube and *D_2_* for the outer tube can be seen from Figure 5.

As shown in Figure 5, increasing the material parameter, *D*, the electromagnetic wave propagation frequencies increase, and the cut-off frequency ($\omega_{cut-off})$ is obtained as 1.883 × 10^8^ Hz.

**Figure 6:**
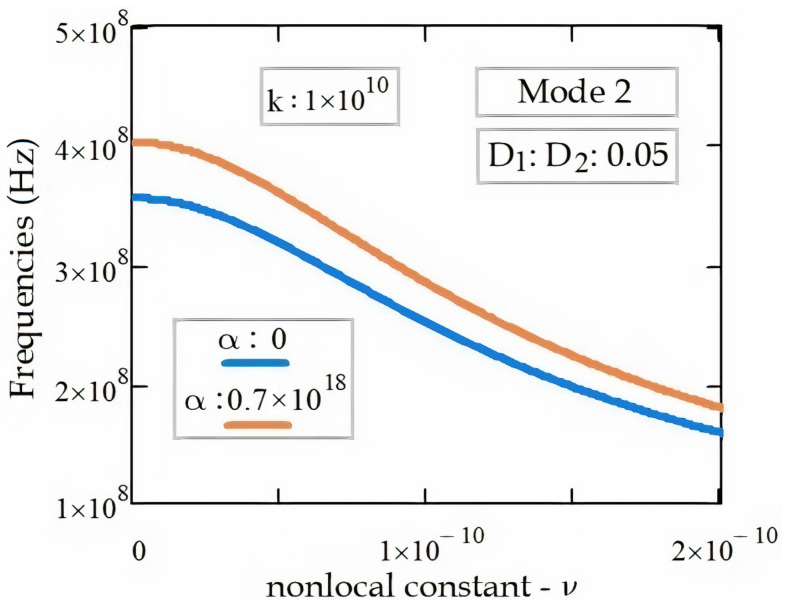
Frequencies of EM wave propagation in the DWCNT for a fixed wavenumber of k = 1 × 10^10^.

The effects of DWCNT’s nonlocal parameter, *ν*, and the DWCNT’s interaction coefficient, α, between the inner and outer tubes on the electromagnetic wave propagation frequencies are shown in Figure 6. For a fixed wavenumber value of k = 1 × 10^10^, the frequencies vary inversely proportional with the nonlocal parameter, ν. Additionally, the frequencies vary directly proportional to the DWCNT’s tubes’ interaction coefficient, α.

**Figure 7:**
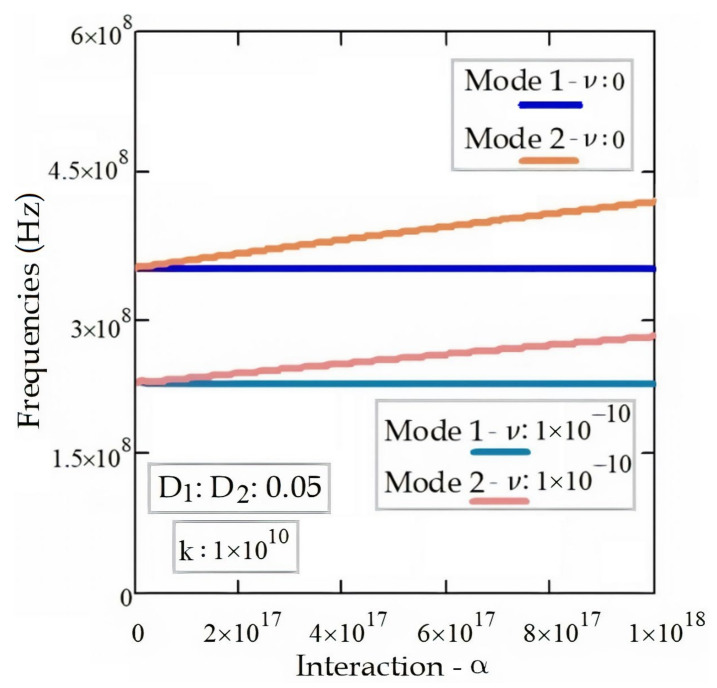
Frequencies of EM wave propagation in the DWT regarding the various interaction coefficients and nonlocal constants for *ν*: 0 and *ν:* 1 × 10^−10^.

Figure 7 depicts the frequencies of electromagnetic wave propagation in the DWT according to the various interaction coefficients, α. As can be seen from the figure, Mode 2 frequencies increase with the different interaction coefficients, α. The Mode 1 frequencies remain constant while the interaction between the tubes increases. In this case, the electromagnetic waves in the tubes of the DWT are in phase. As the interaction between the tubes increases, Mode 2 frequencies also increase. Both Mode 1 and Mode 2 frequencies decrease as the nonlocal constant, *ν*, increases.

**Figure 8:**
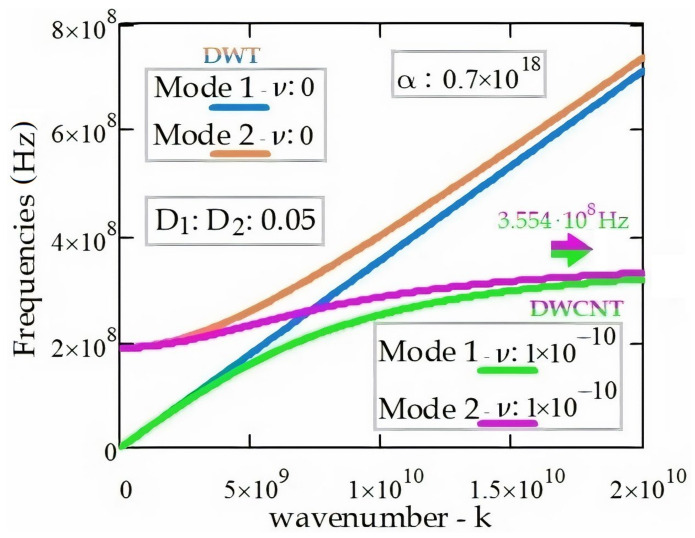
The EM wave dispersion relations (k, ω) related to DWT and DWCNT.

Figure 8 illustrates the behaviors of electromagnetic wave propagation in the DWT and the DWCNT. As can be seen from the figure, the frequencies of electromagnetic wave propagation in the DWCNT are less affected than the frequencies of electromagnetic wave propagation in the DWT from the increase of nonlocal parameter, *ν*. In the DWT, Mode 1 and Mode 2 frequencies of electromagnetic wave propagation converge to each other for high wavenumbers. When the wavenumber, k, is greater than 1.8 × 10^10^, the Mode 1 frequency, $\omega_{1}$ and the Mode 2 frequency, $\omega_{2}$ are obtained as 3.554 × 10^8^ Hz. The electromagnetic wave propagation can occur when cut-off frequency, $\omega_{cut-off}$ is higher than 1.883 × 10^8^ Hz.

**Figure 9:**
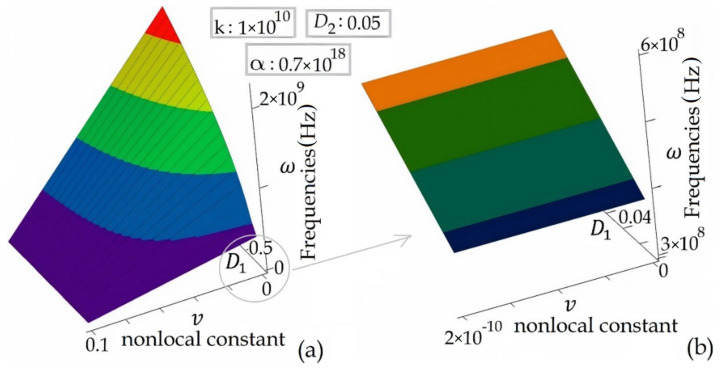
(**a**) The Mode 2 frequencies of EM wave propagation in the DWCNT for the various material parameters, *D*_1_ and the nonlocal constants, *ν*, (**b**) Zoomed version.

Figure 9 depicts the relationship between the Mode 2 frequencies of electromagnetic wave propagation in the DWCNT and DWCNT’s inner tube material parameter, *D_1_* and the nonlocal constant, *ν*. As can be seen from the figure, the electromagnetic wave propagation frequencies increase as the material parameter *D_1_* increases and decreases as the nonlocal constant *ν* increases.

**Figure 10:**
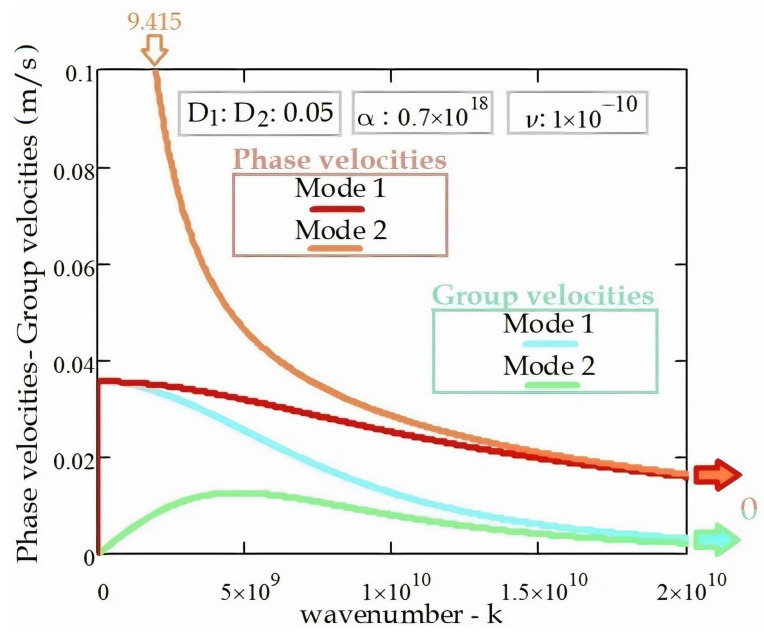
The phase and group velocities of the EM wave propagation in the DWCNT.

Figure 10 illustrates the relationship between the phase velocities (v_phase_) and group velocities (v_group_) for the Mode 1 and Mode 2 frequencies of the electromagnetic wave propagation in the DWCNT. It should be noted that the phase and group velocities decrease by increasing wavenumber. In addition, the interaction coefficient between the DWCNT’s tubes has a reducing effect on the phase and group velocities. As can also be seen from the figure, group velocities increase and then decrease, starting from 0 m/s. For Mode 2 frequencies, the phase velocities decrease, starting from a high value of 9.415 m/s.

## 4. Discussion

Since a SWT comprises a single tube, there is no interaction between the tubes. Therefore, Mode 1 and Mode 2 frequencies of electromagnetic wave propagation are obtained coincidentally, as expected. In case of an interaction between the DWCNT’s inner and outer tubes, Mode 2 frequencies increase starting from a certain cut-off frequency value of 1.884 × 10^8^ Hz. When the *D* material parameter increases, frequencies of the electromagnetic wave propagation in the DWT increase. The interaction between the DWCNT’s tubes affects the Mode 2 frequencies of electromagnetic wave propagation in the DWCNT. In addition, the nonlocal parameter of the DWCNT affects the interaction coefficient between the DWCNT’s tubes. It should be noted that the electromagnetic wave propagation frequencies decrease with the increase of the DWCNT’s nonlocal parameter. The electromagnetic wave propagation frequencies obtained at values greater than 1.5 × 10^−10^ of the nonlocal parameter decrease with a small slope. Mode 1 and Mode 2 frequencies of electromagnetic wave propagation in the DWT, which are obtained for values greater than 2 × 10^9^ of the wavenumber, progress by converging. Mode 1 and Mode 2 frequencies of electromagnetic wave propagation in the DWCNT, for values greater than 1.8 × 10^10^ of the wavenumber proceed in converge to each other. In addition, at values greater than 1.8 × 10^10^ of the wavenumber, Mode 1 and Mode 2 frequencies converge to 3.554 × 10^8^ Hz.

The data obtained in this study show that there is an inverse proportion between the nonlocal parameter and wave propagation frequencies, and it conforms to the general dispersion relation (k, ω) [49,50,51,52,53]. In addition, this study examines the effect of the interaction between the tubes of the DWCNT on wave propagation frequencies, unlike other studies in the literature. The data obtained reveal that there is a direct proportion between the Mode 2 wave propagation frequencies and the electromagnetic interaction coefficient between the tubes of the DWCNT.

The main motivations and novelties of this study are as follows:

- Behaviors of wave propagation have been investigated by some scientists studying nonlocal elasticity in an electromagnetic environment [52,53]. In these studies, the mechanical effects of nanostructures on wave propagation have been primarily investigated. One of the novelties of this study is that behaviors of electromagnetic wave propagation in the DWCNT have been investigated when the DWCNT is only under electromagnetic effect instead of mechanic effect.
- Unlike other studies in the literature, this study reveals electromagnetic wave dispersion relation for Mode 2 frequencies, while the electromagnetic waves propagating in the inner and outer tubes of the DWCNT are in the anti-phase.
- In previous studies, the behaviors of electromagnetic wave propagation in the SWCNT have been examined [54,55,56]. Therefore, in these studies, the effects of electromagnetic interaction between the tubes have not been mentioned. In this study, unlike other studies in the literature, while investigating the behaviors of electromagnetic wave propagation in the DWCNT, both the interaction coefficient between the tubes of the DWCNT and the non-local constant have been taken into account.
- Accompanied by these motivations, the major purpose of this study is to identify the missing key steps in relevant fields and provide data for the benefit of scientists studying theoretically.

## 5. Conclusions

In this article, the behaviors of electromagnetic wave propagation in the double-walled carbon nanotube have been investigated. For this purpose, frequencies of electromagnetic wave propagation in the DWCNT whose inner and outer tubes have different material parameters have been obtained. The effects of both the inner and the outer tubes’ material parameters on the behaviors of electromagnetic wave propagation in the DWCNT have been examined. The effects of the nonlocal parameter on the behaviors of electromagnetic wave propagation in the DWCNT have been mentioned. It has been concluded that the frequencies of electromagnetic wave propagation in the DWCNT can be adjusted with respect to both the interaction coefficient between the tubes of the DWCNT and the nonlocal constant.

In line with the results obtained from this study, the following inferences can be made:

- The frequencies of electromagnetic wave propagation in the DWCNT increase with the increase in the material parameter of the DWCNT’s inner and outer tubes.
- The frequencies of electromagnetic wave propagation in the DWCNT increase with the increase in the interaction coefficient between the tubes of the DWCNT.
- The frequencies of electromagnetic wave propagation in the DWCNT increase with the decreasing slope as the number of waves increases up to a certain frequency limit of 3.554 × 10^8^ Hz. Additionally, the frequencies of electromagnetic wave propagation in the DWT increase with the increasing slope as the number of waves increases.
- The frequencies of electromagnetic wave propagation in the DWCNT decrease with the increase in the nonlocal parameter of the DWCNT.

As a future research, this analysis can also be performed for multi-walled carbon nanotubes (MWCNTs), two-dimensional shells, and plates.

## Data Availability

Not applicable.
